# Rice Defensin OsAFP1 is a New Drug Candidate against Human Pathogenic Fungi

**DOI:** 10.1038/s41598-018-29715-w

**Published:** 2018-07-30

**Authors:** Akihito Ochiai, Kodai Ogawa, Minami Fukuda, Masahiro Ohori, Takumi Kanaoka, Takaaki Tanaka, Masayuki Taniguchi, Yoshiyuki Sagehashi

**Affiliations:** 1 Department of Materials Science and Technology, Faculty of Engineering, Niigata University, Niigata, Japan; 2 Hokkaido Agricultural Research Center, National Agriculture and Food Research Organization (NARO), Hokkaido, Japan

## Abstract

Fungal infections, such as candidiasis and aspergillosis, are some of the most frequent infections in humans. Although antifungal drugs are available for the treatment of these infections, antifungal agents with new mechanisms of action should be developed because of the increasing incidence of drug-resistant pathogens in recent years. In this study, a basic functional analysis of rice defensin OsAFP1, a novel antifungal drug candidate, was conducted. OsAFP1 exerted fungicidal activity against *Candida albicans*, the most common pathogenic fungus in humans, at 4 μM concentration, but it did not inhibit the growth of human pathogenic bacteria. In addition, OsAFP1 retained structural stability after heat treatment at 100 °C for 10 min and after serum treatment at 37 °C for 24 h. A propidium iodide (PI) uptake assay and mutational analysis revealed that amino acid residues within the C-terminal γ-core motif of OsAFP1, particularly Leu-39 and Lys-41, play an important role in its antifungal activity. Further, PI uptake and apoptosis assays suggested that OsAFP1 exerts its antifungal activity by inducing apoptosis of target cells. Immunohistochemistry showed that the OsAFP1 target molecule was located in the cell wall. These findings indicate that OsAFP1 may be developed into a potent antifungal drug.

## Introduction

Fungal infections, such as candidiasis and aspergillosis, are among the most serious infectious diseases. Several tissue conditioners containing various antifungal medicaments, including organic, inorganic, natural, and herbal medicaments, are used to treat these diseases^1^. The following four major groups of antifungal drugs are used as organic medicaments: azoles and allylamines, such as fluconazole and terbinafine, which disrupt cell membranes by inhibiting ergosterol biosynthesis; polyenes, such as nystatin and amphotericin, which cause the destruction of cell membranes and loss of membrane function by binding to ergosterol; and echinocandins, such as caspofungin and micafungin, which cause cell wall damage by inhibiting β-d-glucan synthesis^1–3^. An increase in the incidence of drug-resistant microbes has been recently observed; this is anticipated to possibly pose a serious problem for the treatment of fungal infections^4–6^. Development of antifungal drugs with new mechanisms of action is therefore required to fight such drug-resistant microbes^4^.

Plants produce antimicrobial peptides and proteins, e.g. thionin, defensin, lipid transfer protein, cyclotide, hevein-like protein, and knottin-type peptide, as part of their innate immune response to protect themselves against pathogen-associated infections and stress caused by unfavourable environmental conditions^7,8^. Many of these substances are rich in cysteine residues, which contribute to the stabilization of their structures by disulphide bond formation. Defensins, low-molecular-weight peptides composed of approximately 50 amino acid residues and rich in cysteine and basic amino acids, are produced by animals, insects, and plants^7,9–11^. Because of their broad spectrum of antimicrobial activity against gram-positive and gram-negative bacteria, defensins are regarded as attractive antibacterial agents. The biological functions, structures, and antifungal mechanisms of representative defensins, e.g., human α-defensins, such as HNP1-4 and HD5^12,13^, and β-defensin hBD-1-3^14–16^, have been reported in detail. Many defensins, including these, are well known to play significant roles in the oral cavity, one of the main targets of pathogenic fungi^17^.

The genomic sequence of the *Oryza sativa japonica* (rice) cultivar Nipponbare was determined by the International Rice Genome Sequencing Project (IRGSP) in 2005^18^, and the genome assembly and sequence have since been updated and validated^19^. In the MSU Rice Genome Annotation Project database (http://rice.plantbiology.msu.edu/index.shtml), dozens of defensins have been annotated as defensin and defensin-like (DEFL) family peptides^19^. Among them, those defensins showing high expression levels in seeds, such as DEF1 (Os01g70680), DEF7 (Os02g41904), DEF8 (Os03g03810), and DEFL1 (Os02g07550), were considered to be worthy of selection as drug candidates, considering production efficiency.

In this study, antifungal activity assays against *Candida albicans*, the most common pathogenic fungal species that causes candidiasis in humans^20^, revealed that 4 μM DEF7 completely inhibited its proliferation. In a previous study, DEF7 (designated OsAFP1 in subsequent papers) was reported to exhibit antimicrobial activity against rice pathogenic microbes, such as *Pyricularia oryzae*^21^ and *Helminthosporium oryzae*^22^. Based on this latest knowledge, basic functional analyses of *O. sativa* OsAFP1 were conducted to assess its potency as a novel antifungal drug. Specifically, the antimicrobial spectrum of OsAFP1 against various human pathogenic microorganisms, stability upon heat and serum treatments, mechanism of action, and structural factors responsible for its antifungal activity were analysed.

## Results

### Antimicrobial activity of OsAFP1

**Figure 1:**
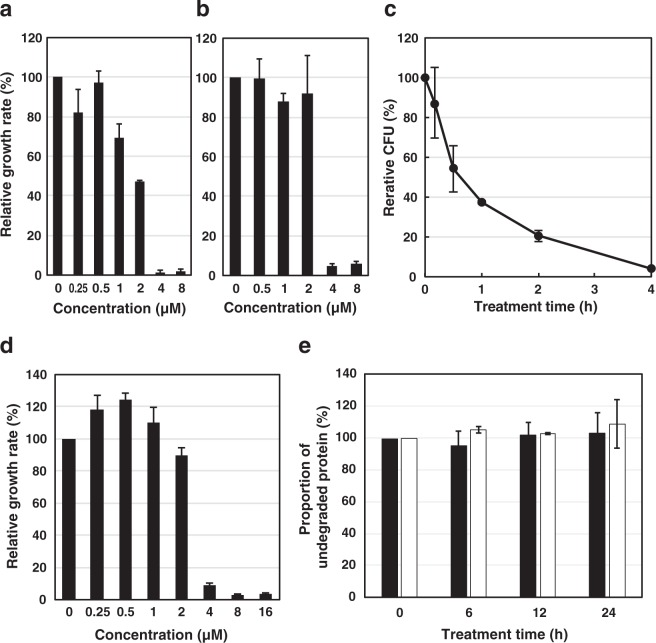
Antifungal activity and stability of OsAFP1. Antifungal activity of OsAFP1 against *C. albicans* CAI4 (**a**) and *S. cerevisiae* BY4742 (**b**) cells. Cells were incubated with different OsAFP1 concentrations, as indicated. After incubation, OD_650_ was measured. (**c**) Fungicidal effect of OsAFP1. *C. albicans* CAI4 cells were incubated with OsAFP1 at 16 μM (4 × MIC) for the time indicated. After incubation, CFUs at each time point were calculated. (**d**) Antifungal activity of OsAFP1 after heat treatment at 100 °C for 10 min. *C. albicans* CAI4 cells were incubated with different concentrations of heat-treated OsAFP1 (0, 0.25, 0.5, 1, 2, 4, and 8 μM). Data for panel (a–d) are presented as the means ± standard deviations (*n* = 3). (**e**) Biodegradation of OsAFP1 after serum treatment. OsAFP1 was incubated with human serum at 37 °C for 0, 6, 12, and 24 h. OsAFP1 degradation at each time point was analysed by Tricine-SDS-PAGE. Data are presented as means ± standard deviation (*n* = 2). The culture conditions and full experimental details are described in the Materials and Methods section.

We cloned the OsAFP1-encoding gene, then produced the recombinant peptide in *Escherichia coli* and purified it. Antifungal activity assays revealed that OsAFP1 significantly suppresses the growth of *C. albicans* CAI4 (IFM 61937) cells in a concentration-dependent manner; the MIC and half maximal inhibitory concentration (IC_50_) were determined to be 4 μM and 2 μM, respectively (Fig. 1a). Next, the antimicrobial activities of OsAFP1 against several human pathogenic gram-negative or gram-positive bacteria and other fungi were examined. OsAFP1 exhibited antifungal activity against two yeast strains, *Saccharomyces cerevisiae* BY4742 (MIC of 4 μM; Fig. 1b) and *S. cerevisiae* S288C (MIC of 16 μM). The defensin, at concentrations up to 32 μM, did not show any growth inhibition against five bacterial strains, namely, *E. coli* K-12, *Porphyromonas gingivalis* ATCC 33277, *Streptococcus mutans* JCM 5705, *Staphylococcus aureus* NBRC 12732, and *Propionibacterium acnes* JCM 6473 (Table 1). This suggested that OsAFP1 is a fungi-specific antibiotic.

**Table 1:** Antimicrobial activity of OsAFP1.

Microbe	MIC (μM)
Fungi	
*Candida albicans* CAI4	4
*Saccharomyces cerevisiae* BY4742	4
*Saccharomyces cerevisiae* S288C	16
Gram-negative bacteria	
*Porphyromonas gingivalis* ATCC 33277	>32
*Escherichia coli* K-12	>32
Gram-positive bacteria	
*Streptococcus mutans* JCM 5705	>32
*Staphylococcus aureus* subsp. *aureus* NBRC 12732	>32
*Propionibacterium acnes* JCM 6473	>32

A subsequent fungicidal assay was performed to evaluate whether OsAFP1 is a fungicidal or fungistatic agent (Fig. 1c). After treatment with OsAFP1, about 50% of *C. albicans* cells died in 30 min, and almost all cells died in 4 h, indicating that OsAFP1 exhibits antifungal activity against target cells in a fungicidal manner.

### Thermal and serum stability of OsAFP1

Because of multiple disulphide bonds, the structures of some cysteine-rich peptides, including defensins, have been reported to be highly rigid, with excellent thermal stability and protease resistance^23,24^. Molecular masses of reduced and oxidized forms of recombinant OsAFP1 ([M + H]^+^) were determined to be 5963.879 and 5956.508 by matrix-assisted laser desorption ionization time-of-flight mass spectrometry (MALDI-TOF/MS) (Fig. S1). Considering that the calculated molecular mass of OsAFP1 is 5963.87, recombinant OsAFP1 is suggested to have four disulphide bonds in its tertiary structure. Therefore, since OsAFP1 is predicted to have high structural stability, the thermal and serum stability of this peptide were evaluated.

First, antifungal activity against *C. albicans* CAI4 was examined using heat-treated OsAFP1. The heat-treated peptide inhibited the growth of *C. albicans* cells in a concentration-dependent manner with a MIC of 8 μM, i.e., a negligible loss of activity even after heating at 100 °C for 10 min (Fig. 1d). Then, the degree of biodegradation of OsAFP1 incubated for up to 24 h in human serum at 37 °C was analysed using Tricine-sodium dodecyl sulphate-polyacrylamide gel electrophoresis (Tricine-SDS-PAGE). No significant degradation of OsAFP1 was observed even upon conditioning in serum for 24 h (Fig. 1e). These results demonstrated that, as anticipated, OsAFP1 possesses extremely high stability upon heat and serum treatment.

### The effect of OsAFP1 on the *C. albicans* cell membrane

**Figure 2:**
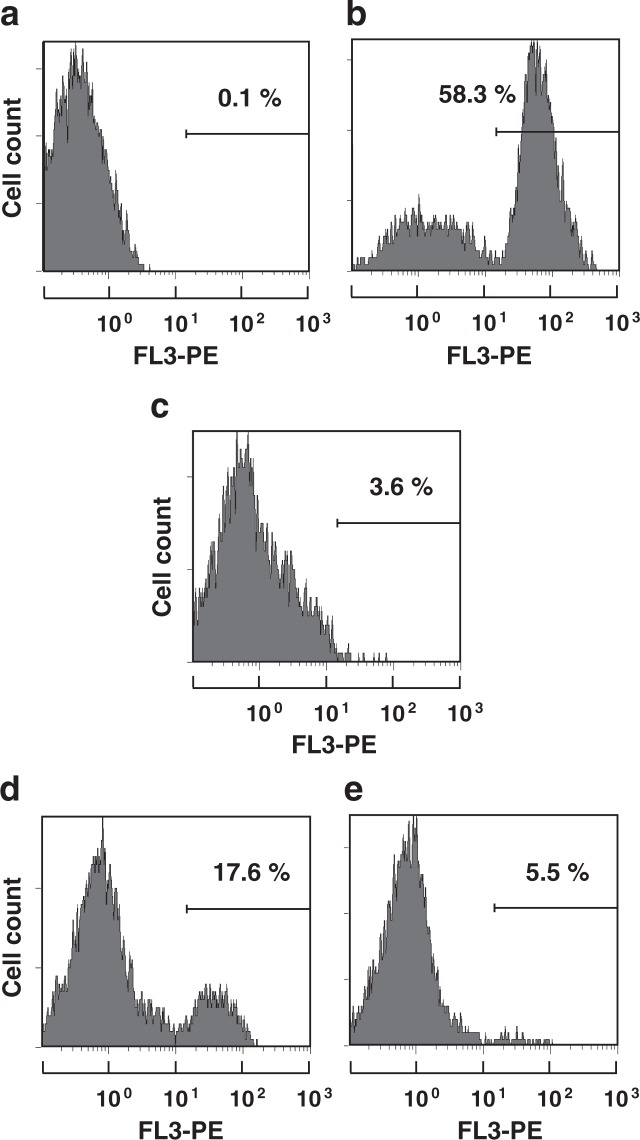
PI uptake assay. *C. albicans* CAI4 cells were treated with PBS (**a**), melittin (**b**), OsAFP1 (**c**), peptide 1 (**d**), or peptide 7 (**e**) at 4 × IC_50_ (6 μM melittin, 8 μM OsAFP1, 20 μM peptide 1, and 40 μM peptide 7) and incubated at 30 °C for 3 h. The PI fluorescence of the cells was analysed by flow cytometry; the cell counts are enumerated in the right field of each histogram. The experimental details have been provided in the Materials and Methods section.

Many antimicrobial peptides and proteins cause cell death by acting on the cytoplasmic membrane of target cells, causing disruption and increased permeation of the membrane^25^. Therefore, the effect of OsAFP1 on *C. albicans* CAI4 cytoplasmic membrane was assessed by a propidium iodide (PI) uptake assay that enabled evaluation of cell membrane permeability by flow cytometry^26^. After treatment with 4 × IC_50_ concentration of melittin, which exerts an antibacterial effect by disrupting the cytoplasmic membrane^27^, 58.3% of cells were stained with PI, indicating that their membranes were disrupted. On the other hand, after OsAFP1 treatment (4 × IC_50_), only 3.6% of cells were stained with PI, suggesting that the peptide does not act on the cytoplasmic membrane to a great extent (Fig. 2a–c).

### Sequence alignment of plant defensins

**Figure 3:**
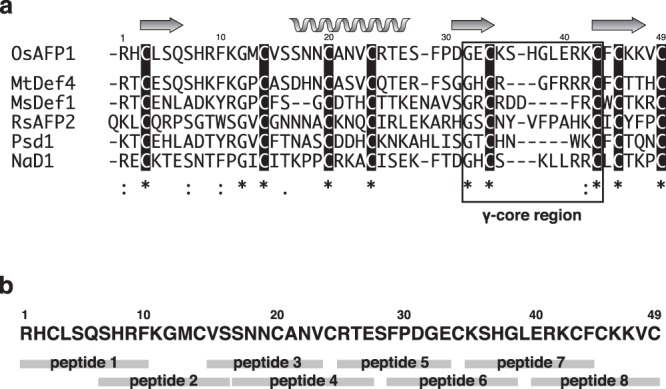
Primary structure of OsAFP1 and design of its partial peptides. (**a**) Alignment of amino acid sequences of plant defensins: OsAFP1 from rice (UniProtKB accession no.: Q6K209); MtDef4 from *M. truncatula* (UniProtKB accession no.: G7L736); MsDef1 from *M. sativa* (UniProtKB accession no.: Q9FPM3); RsAFP2 from *R. sativus* (UniProtKB accession no.: P30230); Psd1 from *P. sativum* (UniProtKB accession no.: P81929); and NaD1 from *N. alata* (UniProtKB accession no.: Q8GTM0). Amino acid sequences were aligned using the Clustal Omega program. Identical, strongly conserved, and weakly conserved amino acid residues are denoted by asterisks, colons, and dots, respectively. Putative secondary structural elements of OsAFP1 are displayed above the sequences. Cysteine residues that are presumed to form disulphide bonds are highlighted in black. The C-terminal γ-core motif is enclosed within a solid box. (**b**) Designed peptides corresponding to partial amino acid sequences of OsAFP1. The peptides correspond to eight overlapping OsAFP1 sequence fragments.

The results of the PI uptake assay with OsAFP1 were not entirely unanticipated. It is well known that many vertebrate and invertebrate defensins (e.g. human HNP1-4 and HD5) exert antibacterial activity by binding to the cytoplasmic membrane and subsequently increasing the membrane permeability of target cells^28–30^. Some plant defensins, such as NaD1 from *Nicotiana alata* and TPP3 from *Solanum lycopersicum*, mainly act by increasing the membrane permeability of their target fungal cells^31–33^. On the other hand, others, such as RsAFP2 from *Raphanus sativus*, MsDef1 from *Medicago sativa*, and MtDef4 from *Medicago truncatula*, not only increase fungal membrane permeability, but also bind to specific components of cell walls or cytoplasmic membranes and exert antifungal activity by inducing apoptosis or disrupting Ca^2+^ signalling and homeostasis^31,32,34,35^. Psd1 from *Pisum sativum* also binds fungal cell walls, induces cell cycle arrest, and blocks K^+^ channels^31,36^. An alignment of the amino acid sequences of these defensins and OsAFP1 is shown in Fig. 3a. As shown, all cysteine residues are well conserved in these defensins; they are presumed to form disulphide bonds as inferred from the three-dimensional structures of NaD1 and MtDef4^37,38^. Furthermore, the γ-core motif (GXCX_3–9_C, boxed in Fig. 3a), reported to be important for antifungal activity of MtDef4^38^, is also conserved. However, other amino acid residues, even those within the γ-core motif, are not conserved in any of the sequences.

### Specific regions of OsAFP1 essential for its antifungal activity

To identify the regions of OsAFP1 associated with antifungal activity, eight overlapping peptides were designed (peptides 1–8). Their amino acid sequences corresponded to different regions of OsAFP1 and collectively covered the entire OsAFP1 sequence; further, each two adjacent peptides overlapped (Fig. 3b). Since MICs could not be determined at a concentration of 25 μM in all the peptides, their antifungal activity was assessed using IC_50_. Antifungal assays revealed that peptide 1 exhibited pronounced antifungal activity; peptides 2, 7, and 8 showed antifungal activity somewhat weaker than that of peptide 1, suggesting that approximately 10 N-terminal and C-terminal amino acid residues are important for the activity (Table 2). *C. albicans* CAI4 PI uptake assays with peptides 1 and 7 (4 × IC_50_) were conducted (Fig. 2d,e). Interestingly, 5.5% of cells emitted fluorescence after treatment with peptide 7, similarly to OsAFP1 treatment, whereas 17.6% of cells emitted fluorescence after peptide 1 treatment. These observations indicated that the mechanism underpinning the antifungal activity of peptide 1 was at least partially different from that of OsAFP1 and did not reflect its properties.

**Table 2:** Antifungal activity of peptides corresponding to partial sequences of OsAFP1 against *C. albicans*.

Peptide	Sequence^*a*^	IC_50_ (μM)
1	RHCLSQSHRF	6
2	SHRFKGMCVS	19
3	VSSNNCANV	>25
4	SNNCANVCRTE	>25
5	RTESFPDGE	>25
6	FPDGECKSHG	>25
7	KSHGLERKCF	10
8	ERKCFCKKVC	13

^a^OsAFP1 regions corresponding to each peptide are shown in Fig. 3b.

Based on the described peptide analysis, the C-terminal region of OsAFP1 was considered to play an important role in its antifungal activity. This was next verified using site-specific mutagenesis. Six amino acid residues in the peptide 7 region of OsAFP1 that had bulky side chains (Lys-35, His-37, Leu-39, Glu-40, Arg-41, and Lys-42) were separately substituted with an alanine residue (yielding OsAFP1 mutants K35A, H37A, L39A, E40A, R41A, and K42A); they were then overproduced in *E. coli* and purified. Because of the low expression levels of the E40A mutant in recombinant *E. coli* cells, the role of Glu-40 in the antifungal activity of the defensin could not be verified. Antifungal activity evaluations of the remaining mutants revealed a large drop in activity (MIC > 32 μM) of all mutant defensins. To compare the reduction of activity of the mutants, IC_50_ values were calculated. The activities of L39A and K41A mutants were so low that the IC_50_ values could not be calculated, in contrast with the remaining mutants (Table 3).

**Table 3:** Antifungal activity of OsAFP1 mutants against *C. albicans*.

OsAFP1 mutant	IC_50_ (μM)
K35A	15
H37A	17
L39A	>32
R41A	>32
K42A	20

Taken together, these results clearly indicated that amino acid residues within the γ-core motif, in particular Leu-39 and Lys-41, play critical roles in the antifungal activity of OsAFP1.

### Apoptosis-inducing effect of OsAFP1

**Figure 4:**
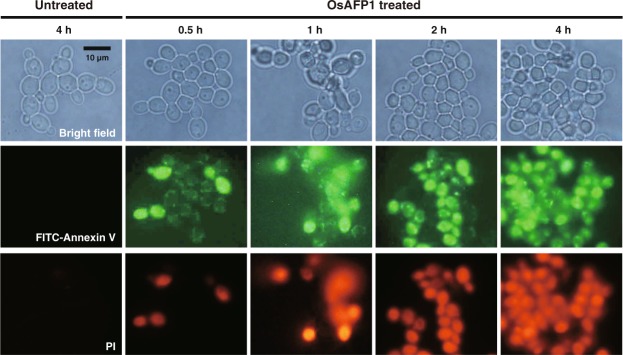
Microscopic observations of *C. albicans* CAI4 apoptotic cells. *C. albicans* CAI4 cells were treated with OsAFP1 at 16 μM (4 × MIC) and incubated at 30 °C for 0, 0.5, 1, 2, and 4 h. At each time point, apoptotic cells were observed under an epifluorescence microscope. *C. albicans* CAI4 cells treated with water and incubated at 30 °C for 4 h was used as a negative control. The top row corresponds to bright field, and the middle and bottom row correspond to dark field microscopic images. The white bars correspond to 10 μm, and the scale is the same in all images. The method is described in detail in the Materials and Methods section.

As described above, the results of the PI uptake experiments indicated that OsAFP1 did not disrupt the plasma membrane of the fungal cells or increase membrane permeability. Therefore, we proposed that the antifungal activity of OsAFP1 might involve the induction of apoptosis (as seen for RsAFP2)^34^ and performed an annexin V assay to detect apoptosis (Fig. 4). Annexin V-FITC, an apoptosis marker, was used to verify this hypothesis. It specifically binds to phosphatidylserine exposed on the apoptotic cell surface in the presence of calcium ions and emits fluorescence^39^, which may be detected using an epifluorescence microscope. Most *C. albicans* CAI4 cells did not emit fluorescence after up to 4 h of treatment with phosphate-buffered saline (PBS), i.e., apoptosis was not induced in those cells. In contrast, almost all fungal cells emitted fluorescence after 0.5 h treatment with OsAFP1, and in particular, FITC fluorescence was detected in the vicinity of the plasma membrane, indicating that early apoptotic morphology was induced. Necrotic cells that only emit PI fluorescence were not detected at any treatment time. In addition, the proportion of dead cells as estimated by PI fluorescence coincided with the results of the fungicidal assay described above. These results suggested that OsAFP1 exhibits antifungal activity by inducing apoptosis of target cells.

### Binding of OsAFP1 on the cell wall

**Figure 5:**
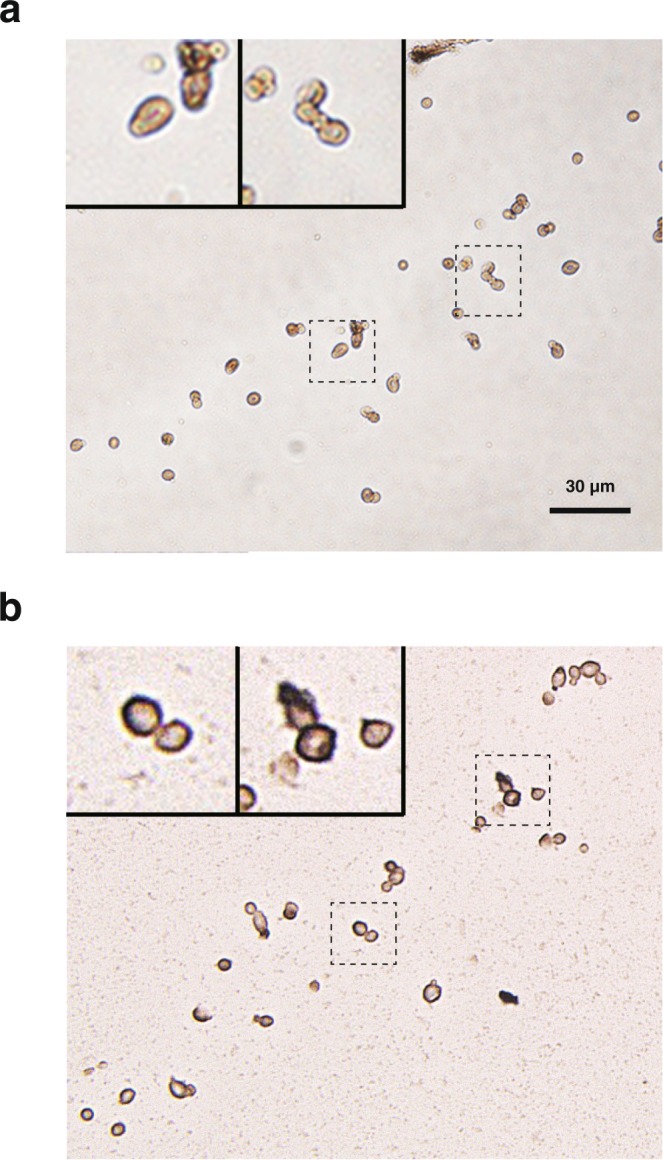
ICC staining of *C. albicans* CAI4 treated with OsAFP1. *C. albicans* cells treated without (**a**) or with (**b**) OsAFP1 were analysed by ICC staining. *C. albicans* CAI4 cells were treated with OsAFP1 at 16 μM (4 × MIC), incubated at 30 °C for 0.5 h, and used for ICC staining. The method is described in detail in the Materials and Methods section.

Immunocytochemical (ICC) staining using an OsAFP1 antibody was performed to identify its target molecule in *C. albicans* cells. The analysis showed that OsAFP1 localised around the cell wall after 0.5 h treatment (Fig. 5). The fungicidal and apoptosis assay described above showed that early apoptosis was induced in all cells 0.5 h after OsAFP1 treatment, while half of the cells were already dead. Taken together, these analyses suggested that OsAFP1 induces apoptosis in *C. albicans* cells by binding to target molecules present around the cell wall, resulting in cell death.

## Discussion

In this study, we systematically examined the antimicrobial properties and mechanism of action of the rice defensin OsAFP1. The peptide is a novel antifungal drug candidate.

Antimicrobial activity assays with various microorganisms, including human pathogenic fungi and bacteria, indicated that OsAFP1 possesses pronounced antifungal activity against *C. albicans* and *S. cerevisiae*, with no activity against the tested gram-negative and gram-positive bacteria. A subsequent fungicidal assay indicated that OsAFP1 exhibits antifungal activity in a fungicidal manner. In addition, OsAFP1 maintained structural stability upon heat treatment (100 °C for 10 min) and serum treatment (at 37 °C for 24 h). These findings indicated that OsAFP1 could be developed into a fungi-specific drug against human pathogens.

Antifungal activity assays, PI uptake assays with eight peptides corresponding to different regions of OsAFP1, and subsequent mutational analysis revealed that the C-terminal region plays an important role in the antifungal activity of this defensin. Peptide 1, corresponding to the N-terminal fragment of OsAFP1, slightly disrupted the membranes of *C. albicans* CAI4 cells; no further mutational analysis of peptide 1 region was performed in this study. Nevertheless, since the effect was negligible, further analysis is required to verify the contribution of the N-terminal region of OsAFP1 in antifungal activity.

**Figure 6:**
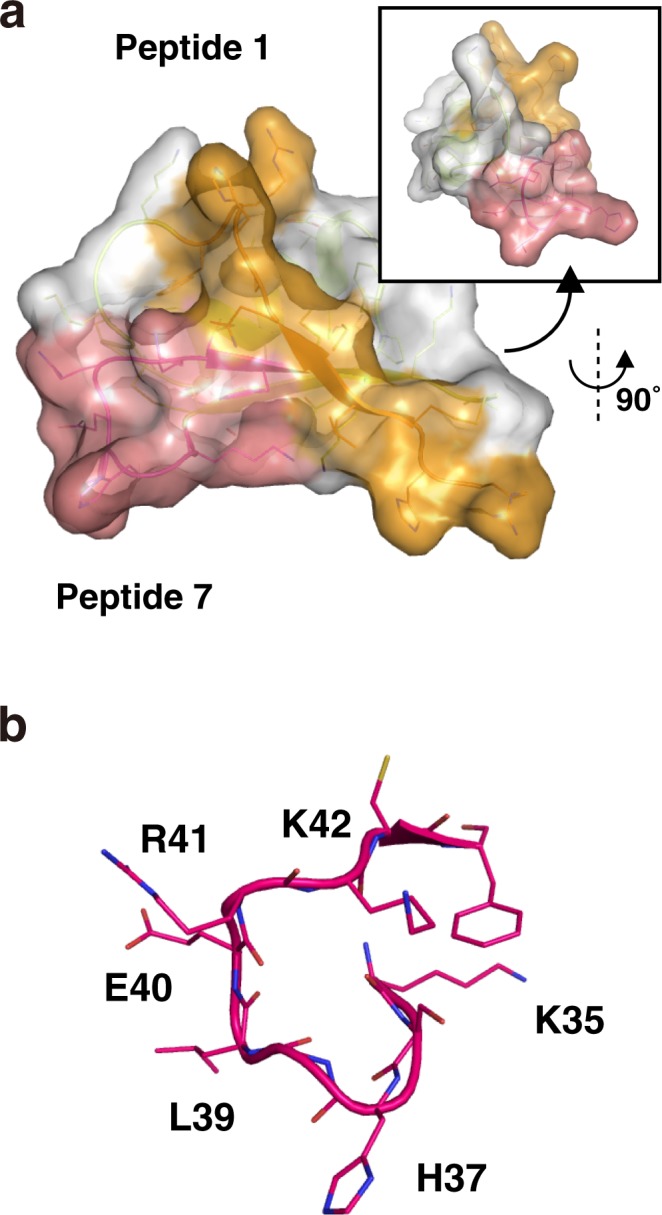
Homology model of OsAFP1. (**a**) The homology model of OsAFP1 generated using the MODELLER program. Detailed parameters are specified in the Materials and Methods section. The surface regions corresponding to peptides 1 and 7 are coloured orange and red, respectively. (**b**) Detailed loop structure corresponding to peptide 7. The atoms of the amino acid residues are designated by different colours, as follows: oxygen, red; nitrogen, deep blue; carbon, magenta; sulphur, yellow.

Our data demonstrated that the γ-core motif, which is preserved in many plant defensins and has been reported to play a role in their antibacterial activity^38,40,41^, is important for the antifungal activity of OsAFP1. Binding to the target molecule has been reported to constitute one of the functions of the γ-core motif. Because most amino acid residues of this motif are not highly conserved, it is reasonable to anticipate that their roles in the γ-core motifs of different defensins differ. Indeed, it has been shown that the ‘^35^RGFRRR^40^’ loop, a region within the γ-core motif of MtDef4, binds to its target molecule, and Phe-37 and Arg-38 are especially important for antifungal activity^41^. In comparison with the MtDef4 γ-core motif, in OsAFP1, two amino acid residues (Ser-36 and His-37) have been inserted, and two (Leu-39 and Glu-40) have been substituted (for Phe-37 and Arg-38 in MtDef4, respectively) (Fig. 3a). Our analysis of OsAFP1 mutants showed that two of these four residues are important for antifungal activity (Table 3). It has been suggested that MtDef4 binds to cytoplasmic phosphatidic acid and kills target cells by destroying Ca^2+^ signalling and homeostasis^38,41^. The above sequence difference in the γ-core motif may be associated with the difference in the mechanisms of action of the two defensins. Furthermore, since RsAFP2, Psd1, and MsDef1 bind to glucosylceramide (GlcCer) and do not exert antifungal activity against GlcCer-lacking strains, it has been suggested that these defensins exhibit antifungal activity by binding to GlcCer in the fungal cell wall^35,42–44^. Since OsAFP1 exhibited antifungal activity against *S. cerevisiae*, which lacks GlcCer^45^ (Fig. 1b and Table 1), it is possible that it induces apoptosis via a target molecule in the fungal cell wall other than GlcCer. ICC staining analysis strongly supported this hypothesis: OsAFP1 was localised around fungal cell walls after treatment. Previously, fluorescent analyses using BODIPY-FL-EDA, FITC, and tetramethyl rhodamine have been applied to some plant defensins, such as NaD1, RsAFP1, and MtDef4^43,46,47^. These analyses also clarify the localization of defensin in the target fungus cell and give strong clues to elucidate its mechanism of action. In the future, we will explore the target of OsAFP1 using a combination of these fluorescent analyses and fungal mutants lacking specific membrane components. The calculated homology model of OsAFP1 is shown in Fig. 6a. The γ-core motif corresponding to peptide 7 is located in a site that likely interacts with the target molecule. In addition, sequence diversity within the motif is considered to contribute to the diversity of the recognized target molecules, as if the motif were a variable region of an antibody (Fig. 6b).

## Conclusion

In the present study, functional analyses of the antifungal activity of the rice-derived defensin OsAFP1 were performed. OsAFP1 completely inhibited the cell growth of *C. albicans* CAI4 at a concentration of 4 μM in a fungicidal manner. Since MIC values of the existing antifungal drugs, such as fluconazole, amphotericin B, and caspofungin, are in the 0.01–1 μM range^48^, further engineering to enhance its function may be required before OsAFP1 can be used as a drug. On the other hand, OsAFP1 was extremely stable upon heat and serum treatments and exerted its antifungal activity by inducing apoptosis, i.e., a mechanism different from that of existing antifungal drugs. These features render OsAFP1 a useful new antifungal drug candidate. Thus, further study of the antifungal mechanism of action of OsAFP1 and verification of its safety towards human cells are warranted.

## Materials and Methods

### Microorganisms and culture conditions

Seven microbial strains were used for OsAFP1 antimicrobial activity testing. *P. gingivalis* ATCC 33277 cells were cultured in modified Gifu anaerobic medium (GAM) (Nissui, Tokyo, Japan) in an anaerobic jar (Mitsubishi Gas Chemical, Tokyo, Japan) in the presence of a deoxygenating regent (AnaeroPack, Mitsubishi Gas Chemical) for 48 h at 37 °C. *E. coli* K-12 NBRC3301, *S. mutans* JCM 5705, *S. aureus* NBRC 12732, and *P. acnes* JCM 6473 cells were cultured in Luria-Bertani (LB) broth^49^, brain-heart infusion broth (Nissui), trypticase soy broth (BD Biosciences, Franklin Lakes, NJ, USA), and GAM broth (Nissui), respectively, under facultative anaerobic conditions for 4–24 h at 37 °C. *C. albicans* CAI4 (IFM 61937) cells were cultured in 0.5 × potato dextrose broth (PDB) supplemented with 25 μg/ml of uridine under facultative anaerobic conditions for 14 h at 30 °C. *S. cerevisiae* BY4742 and S288C cells were cultured in 0.5 × PDB supplemented with 30 μg/ml of uridine, histidine, and lysine and 60 μg/ml of leucine under facultative anaerobic conditions for 14 h at 27 °C. These microbial strains were purchased from the American Type Culture Collection (Manassas, VA, USA), National Institute of Technology and Evaluation (Tokyo, Japan), RIKEN BioResource Center (Tsukuba, Japan), Medical Mycology Research Center (Chiba, Japan), and GE Healthcare (Little Chalfont, UK).

For plasmid amplification, *E. coli* DH5α cells were routinely cultured in LB broth containing ampicillin (100 μg/ml) for 15 h at 37 °C. For the expression of recombinant peptide, *E. coli* Rosetta-gami B (DE3) pLysS cells were aerobically cultured at 30 °C in LB medium supplemented with ampicillin (100 μg/ml) and chloramphenicol (34 μg/ml). When the optical density at 650 nm (OD_650_) reached 0.6, isopropyl-β-d-thiogalactopyranoside was added to the culture at a final concentration of 0.1 mM, and the cells were further cultured at 16 °C for 48 h.

### Antibacterial assays

Recombinant OsAFP1 and its mutants used in this study were prepared as described in “Supplementary Information”. Antibacterial assays to determine the MIC of OsAFP1 were performed using a modified BacTiter-Glo Luminescent cell viability assay kit (Promega, Madison, WI, USA). This assay quantifies ATP in bacterial cultures to determine the number of live cells. All procedures were performed following the manufacturer’s instructions. Briefly, bacterial cells (1 × 10^4^ cells/ml) were cultured in 100 μl of the appropriate medium (as specified in the “Microorganisms and culture conditions” section) in the presence of 0–32 μM OsAFP1 in 96-well Costar cell culture plates. After appropriate incubation for 4–40 h, 10 μl of Lucifell ATP-eliminating reagent (Kikkoman, Chiba, Japan) was added to each well to reduce medium-derived ATP and mixed for 10 min using an orbital shaker. The cell culture (50 μl) was mixed gently with BacTiter-Glo reagent (50 μl) in a well of white Optiplate-96 plates. After incubation for 15 s, luminescence at 560 nm was measured using a plate reader. MIC was defined as the minimum concentration of OsAFP1 that reduced the sample ATP luminescence by >99% compared with that of the control culture. Experiments were independently repeated at least three times.

### Antifungal assays

Antifungal assays to determine the MIC of OsAFP1 were performed as follows. Fungal cells (1 × 10^5^ cells) were suspended in 100 μl of the appropriate medium containing 0–32 μM OsAFP1 in 96-well Costar cell culture plates. After incubation, OD_650_ was measured using a plate reader. MIC was defined as the minimum concentration of OsAFP1 that resulted in >99% reduction of OD_650_ compared with that in the control culture. In some measurements, because of aggregation of medium components, it was difficult to determine MIC values. In those cases, the cultures were plated on agar plates (0.5 × PDB), and the peptide concentration at which viable cell numbers were 1% or less than that in the control culture was considered the MIC. GraphPad Prism version 6.00 for Macintosh (GraphPad Software, La Jolla, CA, USA) was used to calculate IC_50_ values. Experiments were independently repeated at least three times. Detailed growth conditions for each fungus are provided in the “Microorganisms and culture conditions” section above.

### Fungicidal assays

*C. albicans* CAI4 cells (2 × 10^5^ cells/ml) were treated with OsAFP1 and incubated at 30 °C for 0, 0.17, 0.5, 1, 2, and 4 h. After being washed three times with KPB, the cells were plated on agar plates (0.5 × PDB). Calculated CFUs in the plate were used as indicators of viable cells. OsAFP1 was found to exhibit its fungicidal activity with a delayed effect in the course of examining various conditions. Therefore, in the present assay, 4 × MIC concentration was used, because long-term OsAFP1 treatment may produce various undesirable effects other than fungicidal activity and make the assay difficult to interpret. 4 × MIC concentration was also applied in the subsequent annexin V assay and ICC staining analysis.

### Stability assays

Thermal stability of OsAFP1 was evaluated by comparing the MIC of heat-treated (10 min at 100 °C) peptide to the MIC of untreated peptide.

A serum stability assay was used to determine the resistance of OsAFP1 to biodegradation. An OsAFP1 solution (2 μl, 0.9 mM) and 8 μl of human serum (BioWest, Nuaillé, France) were mixed in sterile microtubes; the mixtures were incubated at 37 °C for 0, 6, 12, and 24 h. At each time point, serum proteins were denatured by the addition of equal volumes of 3 M TCA and 6 M urea. In a control experiment, PBS was used instead of human serum, and the mixture was treated as above. After denaturing, the mixtures were centrifuged at 10,000 × *g* for 10 min, and the upper lipid phase was removed. OsAFP1 degradation was analysed by Tricine-SDS-PAGE followed by Coomassie Brilliant Blue staining, as described previously^50^. The peptide band intensity was determined with Image J 1.48^51^, and the time-dependent rate of its degradation was calculated. Experiments were independently repeated at least three times.

### PI uptake assay

*C. albicans* CAI4 cells (2 × 10^5^ cells/ml) were treated with OsAFP1, peptide 1, peptide 7, or melittin at 4 × IC_50_ concentrations (8 μM for OsAFP1, 24 μM for peptide 1, 40 μM for peptide 7, and 6 μM for melittin^52,53^) and incubated at 30 °C for 3 h. After the addition of PI (5 μg/ml) and incubation at room temperature in the dark for 30 min, the cells were analysed using a flow cytometer equipped with a 488 nm air-cooled argon laser. At least 10,000 events per sample were recorded. As a negative control, PBS was used instead of antifungal agents, and the mixtures were treated in the same way.

### Peptide synthesis

Peptides with partial amino acid sequences of OsAFP1 were synthesized using the fluorenylmethyloxycarbonyl solid-phase method and a PSSM-8 automated peptide synthesizer and purified to >98% purity using reversed-phase high performance liquid chromatography with a Cadenza CD-C18 column. Molecular masses of purified peptides were confirmed by MALDI-TOF/MS analysis with an Autoflex III TOF/TOF. Stock solutions of peptides (10 mg/ml) were prepared in sterile ultra-pure water.

### Annexin V assay

*C. albicans* CAI4 cells (2 × 10^5^ cells/ml) were treated with OsAFP1 at 16 μM (4 × MIC) and incubated at 30 °C for 0, 0.5, 1, 2, and 4 h. After being washed three times with KPB, the cells were resuspended in 500 μl of 1 × binding buffer and mixed with 5 μl each of annexin V-FITC and PI reagent (BioVision, Mountain View, CA, USA). The mixtures were incubated at room temperature in the dark for 5 min, and the cells were then observed under a TE2000-S epifluorescence microscope (Nikon, Tokyo, Japan) equipped with an appropriate objective lens [CFI Plan Fluor 100 × oil (Nikon)] and bandpass filters with 542 nm excitation and 620 nm emission for PI and 494 nm excitation and 518 nm emission for FITC.

### ICC staining

The custom rabbit anti-OsAFP1 antibody used in this assay was purchased from Eurofins Genomics (Tokyo, Japan). For ICC staining, *C. albicans* CAI4 cells (2 × 10^5^ cells/ml) were treated with or without OsAFP1 at 16 μM (4 × MIC) and incubated at 30 °C for 0.5 h. The cells were collected, fixed for 2 h in 4% paraformaldehyde phosphate buffer solution, and stored in 1% formalin neutral buffer solution. Sliced sections from each 25 × 35 mm paraffin block containing cells were placed on slides and used for subsequent ICC staining. After washing the slides once with distilled water and three times with PBS, antigen retrieval was carried out in a high-pH antigen retrieval solution (1 mM EDTA, pH 8.0) for 40 min at 95 °C. The slides were kept at room temperature and then immersed in 3% H_2_O_2_ for 5 min to block endogenous peroxidase activity. Sections were treated with anti-OsAFP1 primary antibody (100 × diluted solution) overnight at 4 °C, and then with secondary antibody (Super Sensitive One-Step Polymer-HRP, BioGenex Laboratories, Fremont, CA, USA) for 15 min at room temperature. In between each of the above steps, the sections were washed thrice for 5 min in PBS. The antigen–antibody complex was visualised by diaminobenzidine (Super Sensitive DAB, BioGenex Laboratories) as a chromogen for 1 min at room temperature. The sections were counterstained with Mayer’s haematoxylin solution (Muto Pure Chemicals, Tokyo, Japan) for 1 min, dehydrated, cleared, and mounted.

### Homology modelling of OsAFP1

The homology model of OsAFP1 was generated using the MODELLER program^54^. Three-dimensional structures of γ-thionin from *Hordeum vulgare* (PDB ID: 1GPT), defensin DEF4 from *M. truncatula* (PDB ID: 2LR3), and defensin NaD1 from *N. alata* (PDB ID: 4CQK) were used as template structures. It is presumed that eight cysteine residues in these peptides (and in OsAFP1) are completely conserved and that they have similar tertiary structures (data not shown). The primary structures of OsAFP1 and the template peptides were first aligned using the Clustal Omega program^55^. A homology model of OsAFP1 was then generated by executing a script file in MODELLER based on the alignment; the validity of the structure was verified using the PROCHECK program^56^.

## Electronic supplementary material

Supplementary Information

## References

[1] Iqbal Z, Zafar MS (2016). Role of antifungal medicaments added to tissue conditioners: A systematic review. J. Prosthodont. Res..

[2] Prasad R, Shah AH, Rawal MK (2016). Antifungals: Mechanism of action and drug resistance. Adv. Exp. Med. Biol..

[3] Chen SCA, Sorrell TC (2007). Antifungal agents. Med. J. Aust..

[4] Morschhäuser J (2016). The development of fluconazole resistance in *Candida albicans* - an example of microevolution of a fungal pathogen. J. Microbiol..

[5] Akins RA (2005). An update on antifungal targets and mechanisms of resistance in *Candida albicans*. Med. Mycol..

[6] Sanglard D (1995). Mechanisms of resistance to azole antifungal agents in *Candida albicans* isolates from AIDS patients involve specific multidrug transporters. Antimicrob. Agents Chemother..

[7] Nawrot R (2014). Plant antimicrobial peptides. Folia Microbiol..

[8] Silverstein KA (2007). Small cysteine-rich peptides resembling antimicrobial peptides have been under-predicted in plants. Plant J..

[9] Ganz T (1999). Defensins and host defense. Science.

[10] Lehrer RI (2004). Primate defensins. Nat. Rev. Microbiol..

[11] Otvos L (2000). Antibacterial peptides isolated from insects. J. Pept. Sci..

[12] Greenwald GI, Ganz T (1987). Defensins mediate the microbicidal activity of human neutrophil granule extract against *Acinetobacter calcoaceticus*. Infect. Immun..

[13] Zhao C, Wang I, Lehrer RI (1996). Widespread expression of beta-defensin hBD-1 in human secretory glands and epithelial cells. FEBS Lett..

[14] Bensch KW, Raida M, Mägert HJ, Schulz-Knappe P, Forssmann WG (1995). hBD-1: a novel β-defensin from human plasma. FEBS Lett..

[15] Harder J, Bartels J, Christophers E, Schröder JM (2001). Isolation and characterization of human beta-defensin-3, a novel human inducible peptide antibiotic. J. Biol. Chem..

[16] Harder J, Bartels J, Christophers E, Schröder JM (1997). A peptide antibiotic from human skin. Nature.

[17] Khurshid Z, Zafar MS, Naseem M, Khan RS, Najeeb S (2018). Human oral defensins antimicrobial peptides: a future promising antimicrobial drug. Curr. Pharm. Des..

[18] International rice genome sequencing project. The map-based sequence of the rice genome. *Nature* **436**, 793–800 (2005). 10.1038/nature03895 16100779

[19] Kawahara Y (2013). Improvement of the *Oryza sativa* Nipponbare reference genome using next generation sequence and optical map data. Rice.

[20] Millsop JW, Fazel N (2016). Oral candidiasis. Clin. Dermatol..

[21] Sagehashi Y, Takaku H, Yatou O (2017). Partial peptides from rice defensin OsAFP1 exhibited antifungal activity against the rice blast pathogen *Pyricularia oryzae*. J. Pestic. Sci..

[22] Tantong S (2016). Two novel antimicrobial defensins from rice identified by gene coexpression network analyses. Peptides.

[23] Li CL (2013). Cloning, expression and characterization of antimicrobial porcine beta defensin 1 in *Escherichia coli*. Protein Expr. Purif..

[24] Nguyen PQ (2014). Discovery and characterization of pseudocyclic cystine-knot alpha-amylase inhibitors with high resistance to heat and proteolytic degradation. FEBS J..

[25] Wimley WC (2010). Describing the mechanism of antimicrobial peptide action with the interfacial activity model. ACS Chem. Biol..

[26] O’Brien-Simpson NM, Pantarat N, Attard TJ, Walsh KA, Reynolds EC (2016). A rapid and quantitative flow cytometry method for the analysis of membrane disruptive antimicrobial activity. Plos One.

[27] Chen J, Guan SM, Sun W, Fu H (2016). Melittin, the major pain-producing substance of bee venom. Neurosci. Bull..

[28] Ganz T (2003). Defensins: antimicrobial peptides of innate immunity. Nat. Rev. Immunol..

[29] Lehrer RI, Lu W (2012). α-Defensins in human innate immunity. Immunol. Rev..

[30] Sudheendra US (2015). Membrane disruptive antimicrobial activities of human beta-defensin-3 analogs. Eur. J. Med. Chem..

[31] Cools TL, Struyfs C, Cammue BP, Thevissen K (2017). Antifungal plant defensins: increased insight in their mode of action as a basis for their use to combat fungal infections. Future Microbiol..

[32] Parisi, K. *et al*. The evolution, function and mechanisms of action for plant defensins. *Semin. Cell Dev. Biol*., 10.1016/j.semcdb.2018.02.004 (2018). 10.1016/j.semcdb.2018.02.004 29432955

[33] Payne JA (2016). The plant defensin NaD1 introduces membrane disorder through a specific interaction with the lipid, phosphatidylinositol 4,5 bisphosphate. Biochim. Biophys. Acta.

[34] Aerts AM (2009). The antifungal plant defensin RsAFP2 from radish induces apoptosis in a metacaspase independent way in *Candida albicans*. FEBS Lett..

[35] Muñoz A (2014). Specific domains of plant defensins differentially disrupt colony initiation, cell fusion and calcium homeostasis in *Neurospora crassa*. Mol. Microbiol..

[36] Lobo DS (2007). Antifungal pisum sativum defensin 1 interacts with *Neurospora crassa* cyclin F related to the cell cycle. Biochemistry.

[37] Lay FT, Schirra HJ, Scanlon MJ, Anderson MA, Craik DJ (2003). The three-dimensional solution structure of NaD1, a new floral defensin from *Nicotiana alata* and its application to a homology model of the crop defense protein alfAFP. J. Mol. Biol..

[38] Sagaram US (2013). Structural and functional studies of a phosphatidic acid-binding antifungal plant defensin MtDef4: identification of an RGFRRR motif governing fungal cell entry. Plos One.

[39] Suzuki T (2001). CD24 induces apoptosis in human B cells via the glycolipid-enriched membrane domains/rafts-mediated signaling system. J. Immunol..

[40] De Samblanx GW (1997). Mutational analysis of a plant defensin from radish (*Raphanus sativus* L.) reveals two adjacent sites important for antifungal activity. J. Biol. Chem..

[41] Sagaram US, Pandurangi R, Kaur J, Smith TJ, Shah DM (2011). Structure-activity determinants in antifungal plant defensins MsDef1 and MtDef4 with different modes of action against *Fusarium graminearum*. Plos One.

[42] Thevissen K (2004). Defensins from insects and plants interact with fungal glucosylceramides. J. Biol. Chem..

[43] Thevissen K (2012). The plant defensin RsAFP2 induces cell wall stress, septin mislocalization and accumulation of ceramides in *Candida albicans*. Mol. Microbiol..

[44] Gonçalves S (2012). Evaluation of the membrane lipid selectivity of the pea defensin Psd1. Biochim. Biophys. Acta.

[45] Del Poeta M, Nimrichter L, Rodrigues ML, Luberto C (2014). Synthesis and biological properties of fungal glucosylceramide. Plos Pathog..

[46] Hayes BM (2013). Identification and mechanism of action of the plant defensin NaD1 as a new member of the antifungal drug arsenal against *Candida albicans*. Antimicrob. Agents Chemother..

[47] El-Mounadi K, Islam KT, Hernandez-Ortiz P, Read ND, Shah DM (2016). Antifungal mechanisms of a plant defensin MtDef4 are not conserved between the ascomycete fungi Neurospora crassa and Fusarium graminearum. Mol. Microbiol..

[48] Eksi F, Gayyurhan ED, Balci I (2013). *In vitro* susceptibility of *Candida* species to four antifungal agents assessed by the reference broth microdilution method. *Sci*. World J..

[49] Ausubel, F. M. *et al*. *Current Protocols in Molcular Biology*, John Wiley & Sons, New York, NY., ((eds) 1987).

[50] Schägger H, von Jagow G (1987). Tricine-sodium dodecyl sulfate-polyacrylamide gel electrophoresis for the separation of proteins in the range from 1 to 100 kDa. Anal. Biochem..

[51] Schneider CA, Rasband WS, Eliceiri KW (2012). NIH Image to ImageJ: 25 years of image analysis. Nat. Methods.

[52] Park Y (2004). Design of novel analogues with potent antibiotic activity based on the antimicrobial peptide, HP(2–9)-ME(1–12). Biotechnol. Lett..

[53] Lee DG (2002). HP (2–20) derived from the amino terminal region of helicobacterpylori ribosomal protein L1 exerts its antifungal effects by damaging the plasma membranes of *Candida albicans*. J. Pept. Sci..

[54] Sali A, Blundell TL (1993). Comparative protein modelling by satisfaction of spatial restraints. J. Mol. Biol..

[55] Sievers F (2011). Fast, scalable generation of high-quality protein multiple sequence alignments using Clustal Omega. Mol. Syst. Biol..

[56] Laskowski RA, MacArthur MW, Moss DS, Thornton JM (1993). PROCHECK: a program to check the stereochemical quality of protein structures. J. Appl. Cryst..

