# Supplementary material for: Rice Defensin OsAFP1 is a New Drug Candidate against Human Pathogenic Fungi
Source: Sci Rep. 2018 Jul 30;8:11434. doi: 10.1038/s41598-018-29715-w (PMC6065317; doi:10.1038/s41598-018-29715-w)
Supplement: Supplementary file 1 — Supplementary Information [file 41598_2018_29715_MOESM1_ESM.docx]

**Supplementary Information**

**Rice Defensin OsAFP1 is a New Drug Candidate against Human Pathogenic Fungi**

Akihito Ochiai,^a^* Kodai Ogawa,^a^ Minami Fukuda,^a^ Masahiro Ohori,^a^ Takumi Kanaoka,^a^ Takaaki Tanaka,^a^ Masayuki Taniguchi,^a^ Yoshiyuki Sagehashi^b^*

Department of Materials Science and Technology, Faculty of Engineering, Niigata University, Niigata, Japan^a^; Hokkaido Agricultural Research Center, National Agriculture and Food Research Organization (NARO), Hokkaido, Japan^b^

*Address correspondence to Akihito Ochiai (ottie@eng.niigata-u.ac.jp) and to Yoshiyuki Sagehashi (sagehasi@affrc.go.jp).

**Supplementary Methods**

**Cloning and construction of a plasmid for overexpression of OsAFP1.** The cloning of cDNA and *E. coli* overexpression procedures for the production of recombinant OsAFP1 were similar to methods described previously^1^.

Briefly, genomic DNA from *O. sativa* *japonica* cv. Nipponbare was extracted from leaf tissue using an ISOPLANT kit (Nippongene, Tokyo, Japan). The DNA fragment containing the OsAFP1-encoding gene, encoding the mature peptide without a signal sequence (N-terminal 31 amino acid residues), was amplified by PCR using the following primers: sense, 5′-AGGCACTGCCTGTCGCAGAGC-3′, and antisense, 5′-CTAGCAGACCTTCTTGCAGAAG-3′ and cloned into a pT7Blue vector, yielding pT7Blue/OsAFP1.

The *Bam*HI*-Eco*RI fragment containing the mature OsAFP1-encoding gene was amplified by PCR using the pT7Blue/OsAFP1 as a template and the following primers: sense, 5′-CGCGGATCCAGGCACTGCCTGTCGCAGAGC-3′, and antisense 5′-CCGGAATTCCTAGCAGACCTTCTTGCAGAAG-3′, which harboured *Bam*HI and *Eco*RI restriction sites (underlined). The amplified PCR product was ligated with *Nde*I- and *Xho*I-digested pGEX-6p-1 vector, yielding pGEX-6p-1/OsAFP1; the encoded polypeptide of OsAFP1 was fused with the glutathione *S*-transferase (GST) tag encoded by the vector at the N-terminus. The inserted gene was amplified by PCR using the BigDye™ Terminator v3.1 cycle sequencing kit (Thermo Fisher Scientific, Waltham, MA, USA), and its sequence was then determined by dideoxy-chain termination using an automated DNA sequencer.

**Purification of recombinant OsAFP1.** The recombinant OsAFP1 was produced in *E. coli* cells and purified. Unless otherwise specified, all procedures were carried out at 0–4°C. *E. coli* cells harbouring pGEX-6p-1/OsAFP1 were grown in 375 mL/flask of LB broth containing ampicillin (100 μg/ml) and chloramphenicol (34 μg/ml), collected by centrifugation at 6000 × *g* for 10 min, washed with 20 mM potassium phosphate buffer (KPB) (pH 7.4), and resuspended in the same buffer. Cells were ultrasonically disrupted at 9 kHz for 20 min, and the clear solution obtained by centrifugation at 20,000 × *g* for 20 min was used as the cell extract. The cell extract was applied to a glutathione Sepharose 4 Fast Flow column (1.0 cm × 1.0 cm) equilibrated beforehand with KPB. After washing with 10 ml of KPB, 2 ml of PreScission protease solution [20 U PreScission Protease, 50 mM Tris-HCl (pH 7.3), 50 mM NaCl, and 0.5 mM EDTA] was applied to the column; the protease reaction was performed for 16 h at 4°C. The resultant untagged OsAFP1 was eluted with 10 ml of KPB, and 2 ml fractions were collected every 2 min. The fractions containing OsAFP1 were combined and dialyzed overnight against 20 mM of Tris-HCl (pH 7.5). After dialysis, the sample was applied to a Resource Q column (1.0 cm × 1.0 cm) pre-equilibrated with 20 mM Tris-HCl (pH 7.5). The peptide in the ‘unbound’ flow-through fraction was dialyzed against 20 mM Tris-HCl (pH 7.5) overnight, and the dialysate was used as purified recombinant OsAFP1. The purity and integrity of the purified peptides were determined using Tricine-SDS-PAGE followed by Coomassie Brilliant Blue staining, as described elsewhere^2^. Peptide concentration in the purified fraction was also determined by Tricine-SDS-PAGE analysis using aprotinin, a peptide with a molecular weight equivalent to that of OsAFP1, as a standard. To confirm the molecular weight of purified OsAFP1, MALDI-TOF/MS analysis was performed with an Autoflex III TOF/TOF (Bruker, Billerica, MA, USA). α-Cyano-4-hydroxycinnamic acid was used as the matrix reagent.

**Site-directed mutagenesis of OsAFP1.** To replace Lys-35, His-37, Leu-39, Glu-40, Arg-41, and Lys-42 with Ala residues, OsAFP1 mutants were constructed using a Quik Change site-directed mutagenesis kit (Agilent Technologies, Santa Clara, CA, USA). Plasmid pGEX-6p-1/OsAFP1 was used as a PCR template, and the following oligonucleotides were used as primers: K35A, sense, 5′-CGACGGCGAGTGCGCGTCGCACGGCCTC-3′, and antisense, 5′-GAGGCCGTGCGACGCGCACTCGCCGTCG-3′; H37A, sense, 5′-GCGAGTGCAAGTCGGCCGGCCTCGAGCGC-3′, and antisense, 5′-GCGCTCGAGGCCGGCCGACTTGCACTCGC-3′; L39A, sense, 5′-GCAAGTCGCACGGCGCCGAGCGCAAGTGCTTC-3′, and antisense, 5′-GAAGCACTTGCGCTCGGCGCCGTGCGACTTGC-3′; E40A, sense, 5′-GTCGCACGGCCTCGCGCGCAAGTGCTTC-3′, and antisense, 5′-GAAGCACTTGCGCGCGAGGCCGTGCGAC-3′; R41A, sense, 5′-GTCGCACGGCCTCGAGGCCAAGTGCTTCTGCAAG-3′, and antisense, 5′-CTTGCAGAAGCACTTGGCCTCGAGGCCGTGCGAC-3′; K42A, sense, 5′-CACGGCCTCGAGCGCGCGTGCTTCTGCAAGAAG-3′, and antisense, 5′-CTTCTTGCAGAAGCACGCGCGCTCGAGGCCGTG-3′ (the mutations are underlined). The introduced mutations were confirmed by DNA sequencing, as described above. The OsAFP1 mutants were expressed in *E. coli* and purified using the same procedures as those use to purify wild-type OsAFP1.

**References**

1. Sagehashi, Y. *et al*. Purification and cDNA cloning of a defensin in *Brassica juncea*, its functional expression in *Escherichia coli*, and assessment of its antifungal activity. *J. Pestic. Sci.* **38**, 33–38 (2013).

2. Schägger, H. & von Jagow, G. Tricine-sodium dodecyl sulfate-polyacrylamide gel electrophoresis for the separation of proteins in the range from 1 to 100 kDa. *Anal. Biochem.* **166**, 368–379 (1987).

**Supplementary Figure**

**
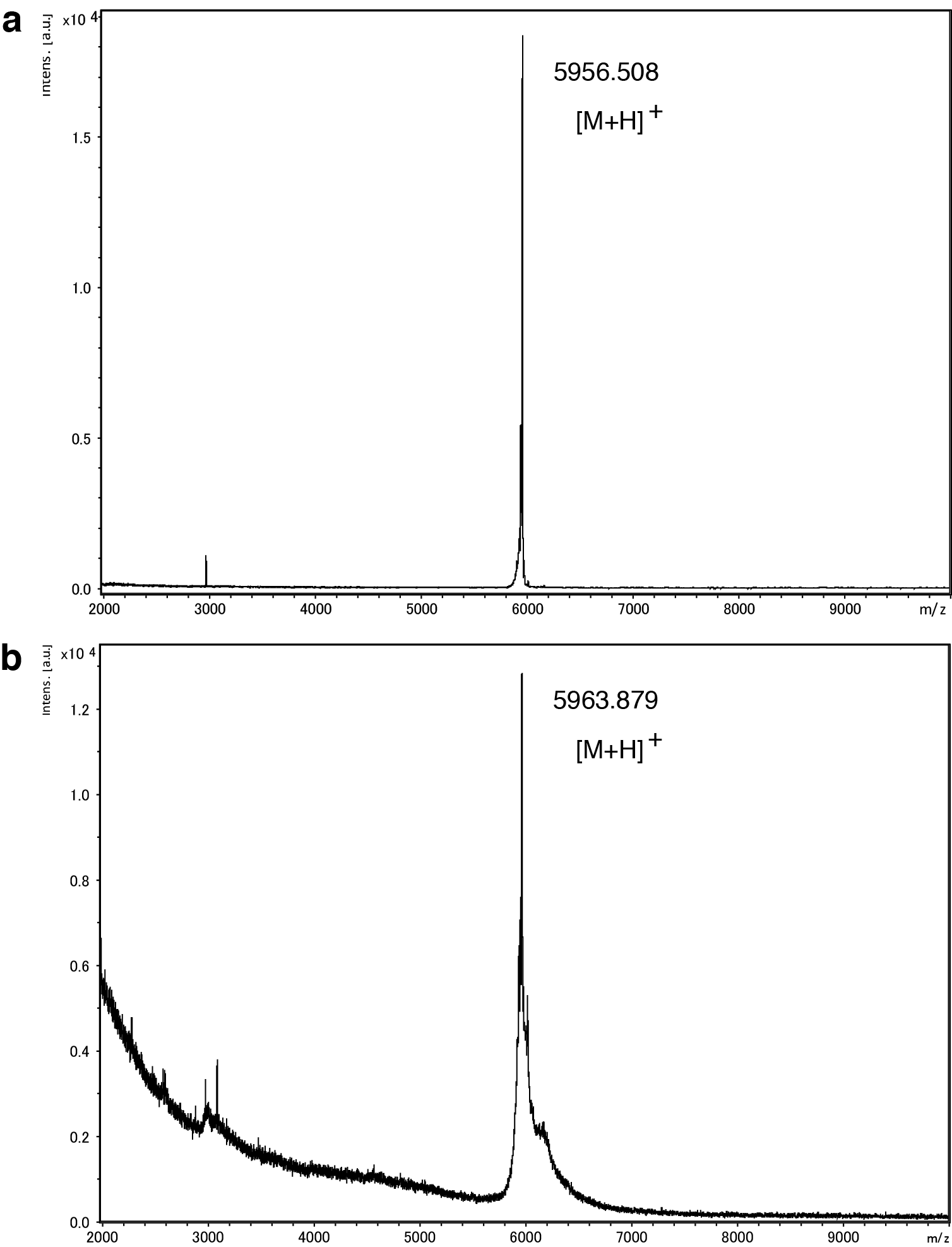
**

**Figure S1.** MALDI-TOF mass spectrums of recombinant OsAFP1 under oxidized (a) and reduced (b) conditions.
